# Reversible Regulation of Catalytic Activity of Gold Nanoparticles with DNA Nanomachines

**DOI:** 10.1038/srep14402

**Published:** 2015-09-23

**Authors:** Peipei Zhou, Sisi Jia, Dun Pan, Lihua Wang, Jimin Gao, Jianxin Lu, Jiye Shi, Zisheng Tang, Huajie Liu

**Affiliations:** 1 Key Laboratory of Laboratory Medicine, Ministry of Education, Wenzhou Medical College, Wenzhou 325035, Zhejiang, China; 2 Division of Physical Biology & Bioimaging Center, Shanghai Synchrotron Radiation Facility, CAS Key Laboratory of Interfacial Physics and Technology, Shanghai Institute of Applied Physics, Chinese Academy of Sciences, Shanghai 201800, China; 3 Department of Endodontics, 9th People’s Hospital, School of Medicine, Shanghai Key Laboratory of Stomatology, Shanghai Jiao Tong University, Shanghai 200011, China

## Abstract

Reversible catalysis regulation has gained much attention and traditional strategies utilized reversible ligand coordination for switching catalyst’s conformations. However, it remains challenging to regulate the catalytic activity of metal nanoparticle-based catalysts. Herein, we report a new DNA nanomachine-driven reversible nano-shield strategy for circumventing this problem. The basic idea is based on the fact that the conformational change of surface-attached DNA nanomachines will cause the variation of the exposed surface active area on metal nanoparticles. As a proof-of-concept study, we immobilized G-rich DNA strands on gold nanoparticles (AuNPs) which have glucose oxidase (GOx) like activity. Through the reversible conformational change of the G-rich DNA between a flexible single-stranded form and a compact G-quadruplex form, the catalytic activity of AuNPs has been regulated reversibly for several cycles. This strategy is reliable and robust, which demonstrated the possibility of reversibly adjusting catalytic activity with external surface coverage switching, rather than coordination interactions.

The fine control of catalytic behavior has long been an interesting topic in chemical and materials sciences^1^. This interest is, in particular, inspired by the growth of our attention and knowledge on enzymes^2^,^3^,^4^. As essentials in metabolism, enzymes are natural biocatalysts that can respond to environmental stimuli to selectively enhance or suppress their catalytic activity. Likewise, the ability to regulate catalytic activity of synthesized catalysts in abiotic systems offers great potential to control the catalytic efficiency and selectivity, as well as to choose catalytic pathway in complex and concurrent processes^5^. Towards this goal, for both natural and synthesized catalysts, a key issue is to regulate the exposing and blocking of active catalytic sites with the help of proper materials. Ligands are widely used for either inducing conformational changes of enzymes^6^,^7^, organometallic and supramolecular catalysts^8^,^9^,^10^, or simply binding to surface active sites of metal nanoparticle catalysts^11^,^12^,^13^. Recently, synthesized nanoparticles^14^ and carbon nanotubes^15^ were also used for adjusting enzymatic activity through surface-binding. However, unlike their natural counterparts, it remains a challenge to regulate the catalytic activity of synthesized catalysts dynamically and especially, reversibly. Though small molecule “effectors” have been demonstrated to be able to switch the catalytic activity of organometallic catalysts through reversible coordination^8^,^9^,^10^, reversibly regulating metal nanoparticles’ catalytic activity is likely to be more difficult.

Herein, we developed a new strategy for circumventing the above problem with DNA nanomachines. With the unparalleled ability of programmable hybridization through unique base pair recognition, DNA has been considered as a powerful material for the construction of functional nanodevices^16^,^17^,^18^,^19^,^20^. Static DNA nanostructures, such as lattices and polyhedrons, are known to be able to anchor functional objects precisely at nanoscale and therefore are ideal platforms for studying distance-dependent interactions^21^,^22^,^23^,^24^. Dynamic DNA nanomachines, on the other hand, can produce nanometer spatial changes driven by external stimuli^25^,^26^,^27^,^28^,^29^. The controllable and reversible features enable DNA nanomachines powerful tools for regulating target binding affinity^30^,^31^, enzyme cascade reaction^32^, and enzymatic activities *in situ*^33^. In this work, we for the first time exploited DNA nanomachines for reversible regulation of catalytic activity of gold nanoparticles (AuNPs). It is important to note that, different from the reversible ligand coordination approach, we utilized the conformational change of a G-quadruplex DNA nanomachine to switch the packing density of the surface-immobilized DNA monolayer on AuNPs, which acts as an adjustable shield for controlling the exposing and blocking of surface catalytic sites.

## Results and Discussion

### Design

**Figure 1:**
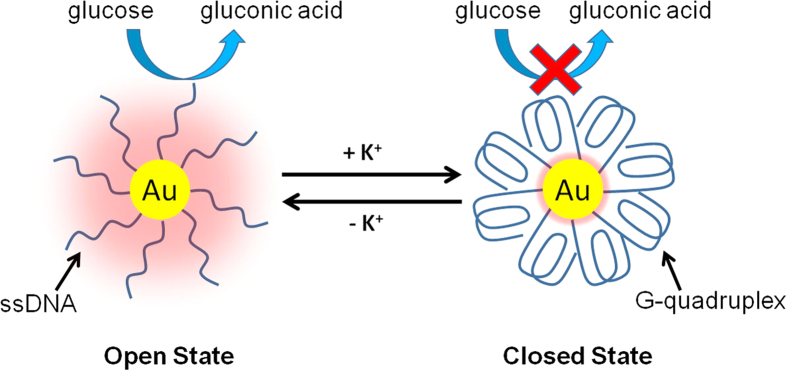
Schematic illustration of the reversible regulation of the GOx-like catalytic activity of AuNPs by G-quadruplex DNA nanomachines.

The working principle of our system is shown in Fig. 1. As one of the most important metal nanoparticle catalysts, AuNPs can catalyze a variety of reactions^34^,^35^. Typically, it is well know that AuNPs have high catalytic activity towards the oxidation of glucose in the presence of O_2_, producing gluconic acid and H_2_O_2_, which mimic natural glucose oxidase (GOx)^36^,^37^,^38^. Though the catalytic mechanism remains elusive, it is believed that the surface gold atoms play essential roles^39^,^40^. Accordingly, the catalytic activity of AuNPs is assumed to be dependent on the degree of their surface coverage with inert materials. It is therefore expected that through adjusting surface coverage on AuNPs, the catalytic activity could be regulated.

G-quadruplex is a special DNA form which is folded quadruply from a G-rich (G = guanine) sequence containing four Gn (n ≥ 2) stretches^41^. A remarkable feature of this structure is its high sensitivity to alkali metal ions, especially K^+^. The conformational change of G-rich sequences driven by K^+^ has been widely used as a model DNA nanomachine due to its simplicity and robustness^25^. Though DNA has been successfully anchored on GOx-mimicking AuNPs recently^42^,^43^,^44^,^45^, studies on G-quadruplex have not been reported. In our design, a thiol-modified G-rich sequence containing four GGG stretches is firstly immobilized on AuNPs (d = 10 nm) surface through thiol-Au bonds. In the absence of K^+^ (open state), these G-rich strands maintain flexible single-stranded conformations (ssDNA) and if the inter-strand distance is large enough, the glucose molecules are expected to be able to interact with surface gold atoms and the reaction can go ahead. In order to alter the activity, K^+^ will then be introduced and under its action, each G-rich strand will fold into a G-quadruplex conformation (closed state) which has a theoretical diameter of 2.5 nm^41^. Since the diameter of a G-quadruplex is much bigger than that of an ssDNA strand, the effective surface coverage in the closed state will be higher than that in the open state, resulting in decreased contact areas between catalyst AuNPs and substrate glucose molecules in the closed state, and thus the deactivation of the catalyst. The catalytic activity of AuNPs is expected to be recovered after removing K^+^, while the DNA nanomachines will transform from the compact closed state back to the flexible open state. Through alternately adding and removing K^+^, these DNA nanomachines will switch between “open” and “closed” states, resulting in a reversible variation of the exposed surface area of AuNPs, and thus achieving a reversible regulation of their catalytic activity.

### GOx-like catalytic activity of DNA-modified AuNPs

Given the assumption of the surface coverage-dependent catalytic activity, the amount of DNA nanomachines should be optimized to avoid low activity caused by high density immobilization and low adjustability caused by low density immobilization. Therefore, we quantitatively studied the effect of ratio between AuNPs and DNA strands on the catalytic activity. According to the reported procedure^46^, naked AuNPs (Fig. S1) were firstly capped with protector dipotassium bis(p-sulfonatophenyl) phenylphosphane dehydrate (BSPP) to increase their stability in ionic solution. Varied amount of thiolated ssDNA strands were then incubated with BSPP-AuNPs to immobilize DNA nanomachines on AuNPs surface. The ratio between AuNPs and DNA strands has been adjusted from 1:1 to 1:100. Agarose gel electrophoresis results demonstrated the success of the immobilization (Fig. S2).

**Figure 2:**
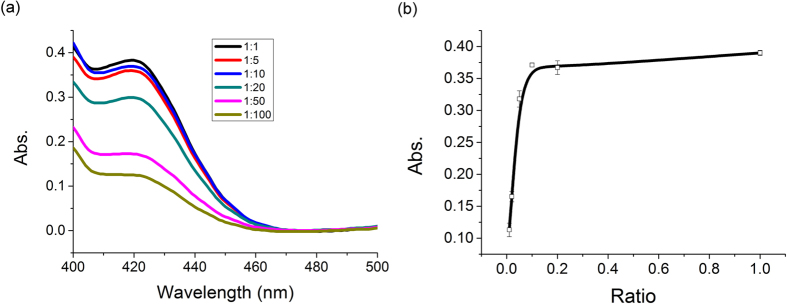
Effect of AuNPs:DNA ratio on the catalytic activity of AuNPs: UV spectra (a) and plot of absorption values at 420 nm (b).

The catalytic activities of as-prepared DNA-AuNPs conjugates were then evaluated with a coupled chromogenic reaction, in which the produced H_2_O_2_ can oxidize 2,2′-azinobis(3-ethylbenzthiazoline-6-sulfonate acid) (ABTS^2−^) and generate characteristic color (max. absorption at 420 nm) in the presence of Horseradish peroxidase (HRP). It is noteworthy that BSPP protected AuNPs have no catalytic activity (Fig. S3), possibly due to the surface passivation. However, as long as AuNPs were functionalized with DNA, they showed the capacity of glucose oxidization (Fig. 2). The catalytic activity kept relatively similar to each other when AuNPs were functionalized with DNA at low density (AuNPs:DNA = 1:1, 1:5, 1:10), but it decreased with further gradually increasing of DNA immobilization density to 1:50 and 1:100. Since the immobilization of thiolated ssDNA could substitute a number of BSPP molecules, a reasonable explanation is that at low density DNA immobilization, the surface area around DNA strand is exposed directly to solution and the catalytic ability has been activated. However, when continuously increasing the amount of thiolated DNA, there would be a competition between two factors. The positive one is that more BSPP molecules are replaced by thiol groups to activate AuNPs. The negative one is probably caused by the increased packing density of ssDNA on AuNPs, as well as the increased non-specific adsorption. In the cases of high density DNA immobilization, the negative factor may play the dominant role and cause the deactivation of AuNPs. From the above test, an optimized AuNPs:DNA ratio of 1:10 has been selected for the subsequent experiments. The catalysis behavior of this conjugate has been analyzed (Fig. S4) and the Michaelis-Menten constant (*Km*) was calculated to be 28.59 mM, showing lower affinity to glucose than naked AuNPs^39^.

### Reversible regulation of catalytic activity of AuNPs

**Figure 3:**
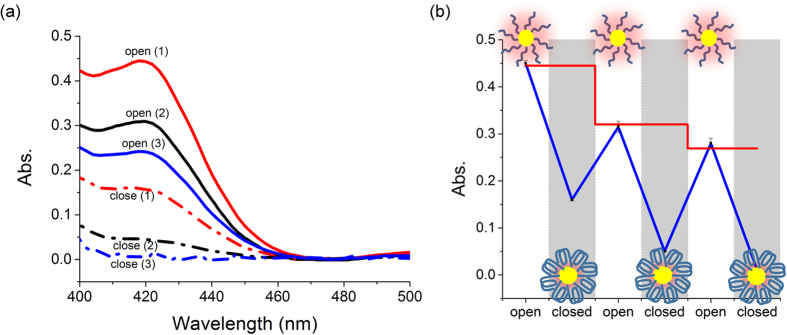
Reversible regulation of the GOx-like catalytic activity of AuNPs: UV spectra (a) and cyclic switching indicated by monitoring the absorption values at 420 nm (b).

To validate our design, we next studied the reversible regulation of the GOx-like catalytic activity with K^+^. From circular dichroism (CD) measurements (Fig. S5), it has been proved that the G-rich sequence in solution exhibited a typical G-quadruplex conformation^47^ in the presence of as low as 5 mM K^+^. G-quadruplex could form completely when 10 mM K^+^ ions was used and this concentration was then applied to induce the conformational change of the G-rich strands on AuNPs. As expected, this led to a remarkable drop in the catalytic activity, which was confirmed with UV absorption characterizations (Fig. 3a and blue curve in Fig. 3b). In order to rule out the possibility that the drop was simply caused by the variation of the K^+^ concentration rather than the K^+^ induced DNA conformational change, control experiments utilizing a random DNA sequence that is non K^+^ responsive as well as some analogous sequences to the used G-rich strand have been carried out. These sequences have a same length with the used G-rich sequence and 2–4 G bases were substituted with T bases in analogous sequences. CD experiments demonstrated that all these sequences can not form G-quadruplex with K^+^, even for the analogous sequences (Fig. S6). To test their influences on the catalytic activity of AuNPs, for each sequence, same amount of DNA has been immobilized on AuNPs to prepare a control DNA-AuNPs conjugate. Under same conditions, all these control conjugates showed almost same catalytic activities with DNA nanomachine-functionalized AuNPs in the absence of K^+^, implying that the sequences themselves had no influence on the catalytic ability. On the other hand, as expected, the catalytic activity remained constant in the presence of K^+^ even if its concentration has been raised to 100 mM (Fig. S7). This test verified the design that the catalytic activity of AuNPs is regulated by the spatial conformation of G-quadruplex formed under the action of K^+^. Furthermore, sodium ions, which were also reported to be important for some G-quadruplex structures^41^, can not help the G-rich sequence to form G-quadruplex and affect the catalytic activity (Fig. S8).

Multiple cycling of the regulation has also been achieved through alternately adding and removing K^+^. However, we noted that the catalytic capacity gradually decreased with each cycle. A control experiment was therefore designed for the purpose of elucidating decay mechanism in this system. Using previously designed non K^+^ responsive control sequence, the control DNA-AuNPs conjugates were tested with the same cycling of adding and removing K^+^. Having run same number of cycles, the catalytic activity of control conjugates definitely decreased (red curve in Fig. 3b). Especially, the degree of the decay after each cycle was in good correspondence with that of G-rich DNA-functionalized AuNPs. According to literatures^39^,^40^, the main product of this reaction, that is gluconic acid, is likely to absorb on AuNPs and induce surface passivation which could be regarded as catalyst poisoning. In addition, the loss of AuNPs after repeated use may be another reason and it has been quantitatively assessed by UV absorption characterization (Fig. S9). We found that the rate of AuNPs loss was slower than that of the decay of the catalytic activity, proving that the decay was partially due to the loss of AuNPs. Though catalyst poisoning and AuNPs loss happened, this experiment clearly demonstrated that the catalytic activity of AuNPs can be reversibly regulated with DNA nanomachines, confirming the effectiveness of the design.

### DLS measurements

**Figure 4:**
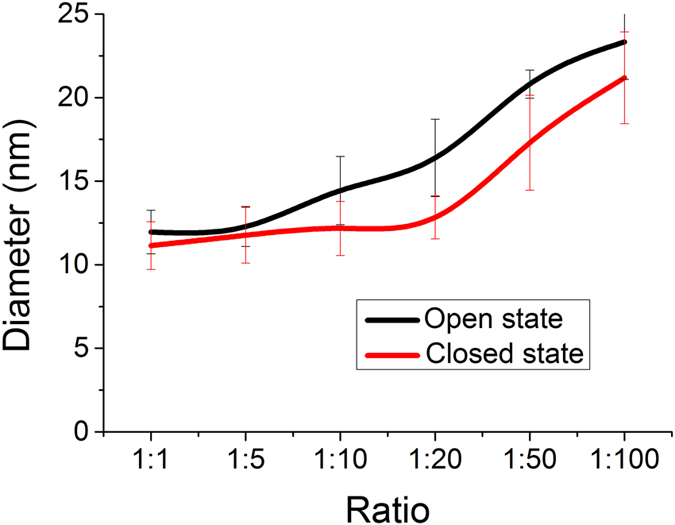
Diameters of DNA-AuNPs conjugates in the open and closed states with different AuNPs:DNA ratios.

Finally, we carried out dynamic light scattering (DLS) measurements to further prove the conformational changes of the DNA nanomachines on AuNPs induced by K^+^, which could be reflected from the change of the hydrodynamic size of AuNPs. Since G-quadruplex conformation is more compact than ssDNA, the size of AuNPs in the closed state was expected to be smaller than that in the open state. As shown in Fig. 4, when the immobilization density of the G-rich DNA on AuNPs was low (AuNPs:DNA = 1:1, 1:5), the measured size of AuNPs in open states (d = ~12 nm) was slightly bigger than that in closed states (d = ~11 nm), which are both close to the theoretical size of naked AuNPs of 10 nm. However, with further increasing the DNA loading amount, the size of AuNPs in open states varied much with that in closed states. The decreased 3–4 nm in diameter was in good agreement with the design and can be attributed to the conformational change from the extended ssDNA to the compact G-quadruplex. Furthermore, the gradual increase of the size with DNA immobilization amount suggested that more and more DNA strands have been densely packed on AuNPs with the result of blocking more surface active sites, which is consistent with the previously performed catalytic activity tests.

## Conclusions

In summary, we developed a simple and reliable strategy for the reversible regulation of the catalytic activity of AuNPs, which was demonstrated with a glucose oxidation reaction as a proof-of-concept study. Different from the reversible ligand coordination approaches used mostly for enzymes and organometallic catalysts, this conceptually new strategy demonstrated the possibility of implementing DNA nanomachines for regulating the catalytic activity of metal nanoparticles. Importantly, the idea used here was based on the fact that the conformational change of DNA nanomachines on surface could lead to a reversible nano-shield^48^,^49^, a phenomenon which has the advantages of fast response, structural robustness and easy control, and has not been applied to catalysis regulation before. Beside AuNPs, it is believed that this strategy is also feasible for other metal nanopartiles, even other catalysts such as enzymes. Since the regulation is driven by surface-attached DNA strands, the conformational switch of catalyst itself could be avoided and the potential harm for irreversible deactivation could be diminished. Given these advantages, it is expected that this strategy will bring new ideas to traditional catalysis and, with further qualitative and quantitative studies, will promote the production of new materials.

## Methods

### Materials

All chemicals and enzymes were purchased from Sigma-Aldrich and used as received. Colloidal solution of 10 nm AuNPs was purchased from BBInternational. DNA sequences were synthesized by Takara Biotechnology Co. and purified with HPLC.

G-rich DNA G4: 5′HS-TTTTTGGGTAGGGCGGGTTGGGTTCGACAGCT-3′

4T: 5′HS-TTTTTGTGTAGTGCGTGTTGTGTTCGACAGCT-3′

3T: 5′HS-TTTTTGTGTAGTGCGTGTTGGGTTCGACAGCT-3′

2Ta: 5′HS-TTTTTGGGTAGTGCGTGTTGGGTTCGACAGCT-3′

2Tb: 5′HS-TTTTTGTGTAGGGCGGGTTGTGTTCGACAGCT-3′

Random control: 5′-GCGTTGCGGAGTGACTGCATTAGAGTCTTTTT-3′SH

### BSPP capping on AuNPs

BSPP (15 mg) was added to the colloidal nanoparticles solution (50 mL) and the mixture was shaken overnight at room temperature. Sodium chloride (solid) was added slowly to this mixture while stirring until the color changed from deep burgundy to light purple. The resulting mixture was centrifuged at 3000 rpm for 30 min and the supernatant was carefully removed with a pipette. AuNPs were then resuspended in 1 mL solution of BSPP (2.5 mM). Upon mixing with 1 mL methanol, the mixture was centrifuged, the supernatant was removed and the product was resuspended in 1 mL BSPP solution (2.5 mM). The concentration of the AuNPs was estimated from the optical absorbance at 520 nm.

### Preparation of AuNPs-DNA conjugates with thiol-modified DNA

The thiolated DNA strands were first reduced by tris(2-carboxyethyl)-phosphine (TCEP) in water and subsequently purified using a G-25 column (GE Healthcare) to remove small molecules. Then thiol-modified oligonucleotides were mixed with BSPP-AuNPs at certain ratio in 0.5 × TBE buffer containing NaCl (50 mM) for 40 h at room temperature. AuNPs-DNA conjugates were washed with 0.5 × TBE buffer using centrifuge filters with a 100 kDa MWCO to remove the extra oligonucleotides. The concentration of conjugates was estimated from the optical absorbance at approximately 520 nm. AuNP-DNA conjugates with discrete numbers of oligonucleotides were characterized by 1% agarose gel (running buffer 0.5 × TBE, loading buffer 50% glycerol, 15 V/cm).

### Glucose oxidation reaction and colorimetric measurements

10 nM AuNPs-DNA conjugates in different ratio were first incubated with 100 mM glucose (in 10 mM Tris-HCl, pH 7.4) at 37 °C for 30 min. Then the mixture was centrifugated at 14,000 rpm for 30 min at 4 °C to remove AuNPs, avoiding the interference of AuNPs in colorimetric reaction. Then horseradish peroxidase (HRP) and 2,2′-azinobis(3-ethylbenzthiazoline-6-sulfonate acid) (ABTS^2−^) were added to the solution to final concentrations of 20 nM and 0.5 mM, respectively. The resulting mixture was further incubated for another 5 min at room temperature and the catalytic oxidation of ABTS^2−^ was monitored at λ = 420 nm with a Hitachi U-3010 UV-Vis spectrophotometer.

In order to regulate the catalytic activity with K^+^, concentrated KCl solution was added into the mixture to a final concentration of 10 mM. To remove K^+^, the mixture was centrifugated at 14,000 rpm for 30 min at 4 °C and then washed with deionized water for 3 times. The product was resuspended in glucose solution for the next catalysis. For size distribution measurements, a Zetasizer μV (Malvern Instruments Ltd.) was used.

## Additional Information

**How to cite this article**: Zhou, P. *et al.* Reversible Regulation of Catalytic Activity of Gold Nanoparticles with DNA Nanomachines. *Sci. Rep.* **5**, 14402; doi: 10.1038/srep14402 (2015).

## Supplementary Material

Supplementary Information
