# Supplementary material for: Reversible Regulation of Catalytic Activity of Gold Nanoparticles with DNA Nanomachines
Source: Sci Rep. 2015 Sep 23;5:14402. doi: 10.1038/srep14402 (PMC4585782; doi:10.1038/srep14402)
Supplement: Supplementary Information [file srep14402-s1.doc]

Supplementary information for

**Reversible Regulation of Catalytic Activity of Gold Nanoparticles with DNA Nanomachines**

Peipei Zhou,^1,2^ Sisi Jia,^2^ Dun Pan,^2^ Lihua Wang,^2^ Jimin Gao,^1^ Jianxin Lu,^1^ Jiye Shi,^2,3^ Zisheng Tang,^3*^ Huajie Liu^1*^

1. Key Laboratory of Laboratory Medicine, Ministry of Education, Wenzhou Medical College, Wenzhou 325035, Zhejiang, China
2. Division of Physical Biology & Bioimaging Center, Shanghai Synchrotron Radiation Facility, CAS Key Laboratory of Interfacial Physics and Technology, Shanghai Institute of Applied Physics, Chinese Academy of Sciences, Shanghai 201800, China
3. Department of Endodontics, 9th People’s Hospital, School of Medicine, Shanghai Key Laboratory of Stomatology, Shanghai Jiao Tong University, Shanghai 200011, China

*Corresponding Authors. Email: [tangzisheng163@163.com](mailto:tangzisheng163@163.com), [liuhuajie@sinap.ac.cn](mailto:liuhuajie@sinap.ac.cn)

**Figures**


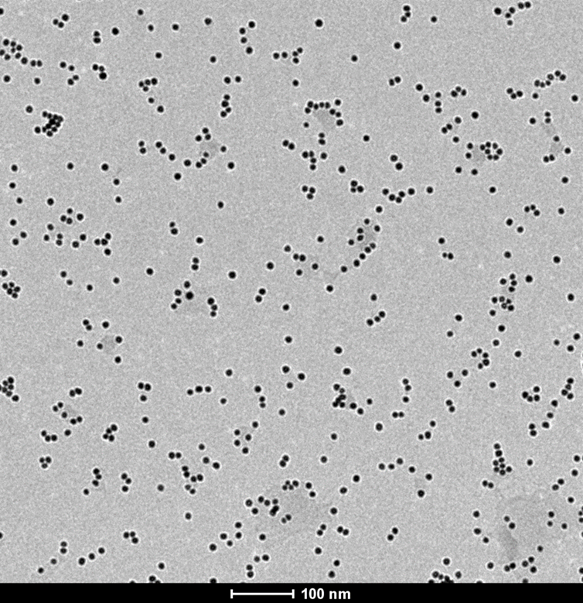


**Figure 1.** TEM image of the 10 nm gold nanoparticle.


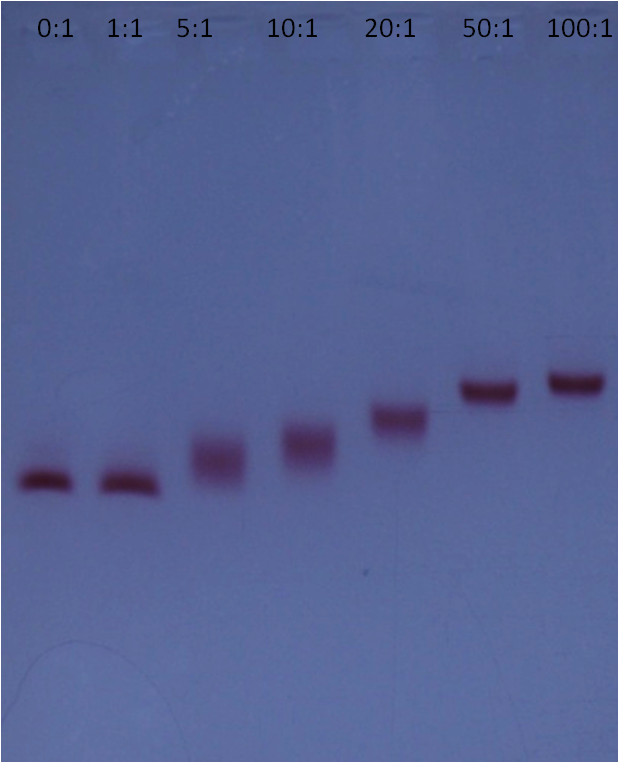


**Figure 2.** Agarose gel electrophoresis showing the AuNP-DNA conjugates with different DNA:AuNPs ratios.


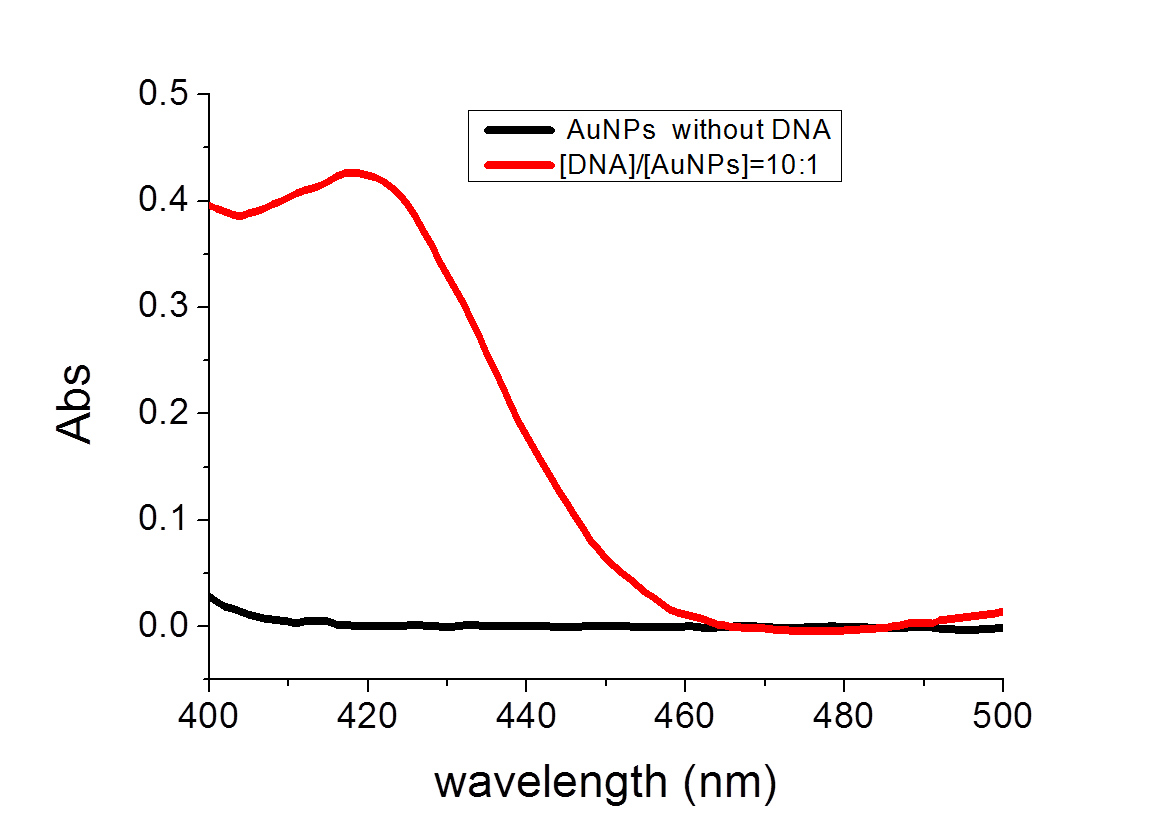


**Figure 3.** Absorption spectra of the colorimetric product in the AuNPs and HRP cascade reaction. Black line: BSPP capped AuNPs; red line: DNA:AuNPs = 10:1.


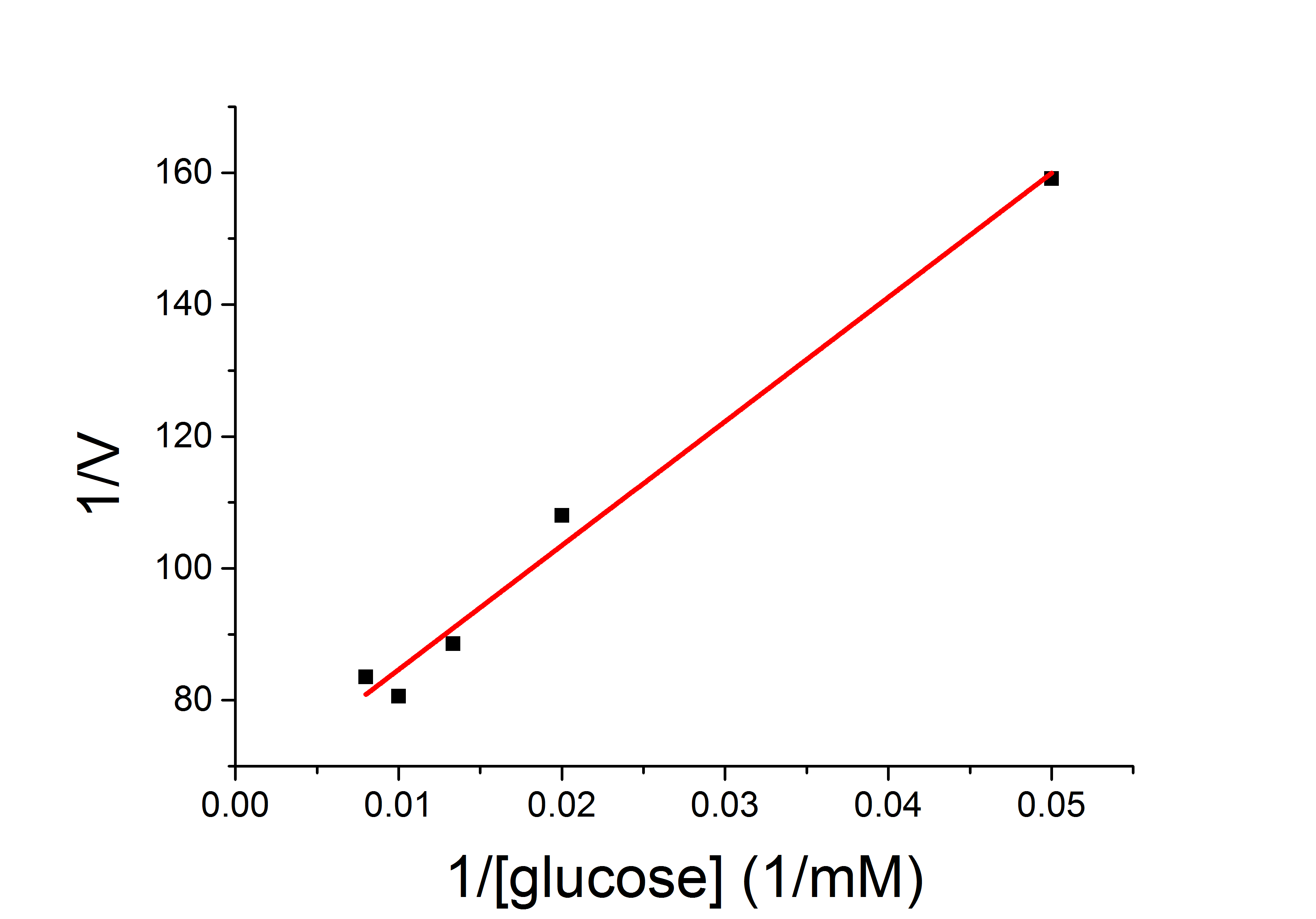


**Figure 4.** Lineweaver-Burke plot of the G-rich DNA capped AuNPs (DNA:AuNPs = 10:1).

**
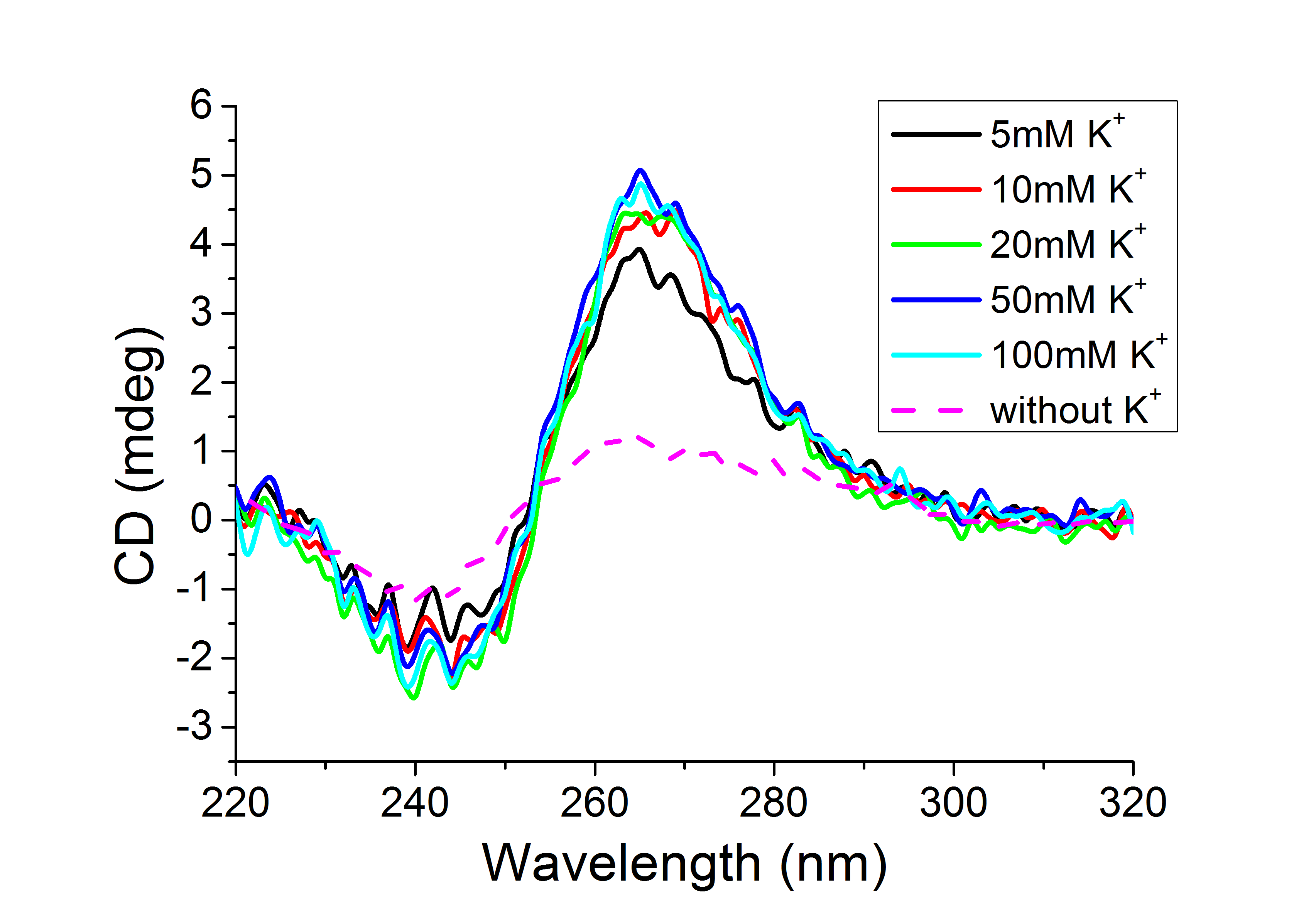
**

**Figure 5.** Circular dichroism (CD) spectra of the G-rich DNA in the presence (solid lines) and absence (dashed line) of K^+^.


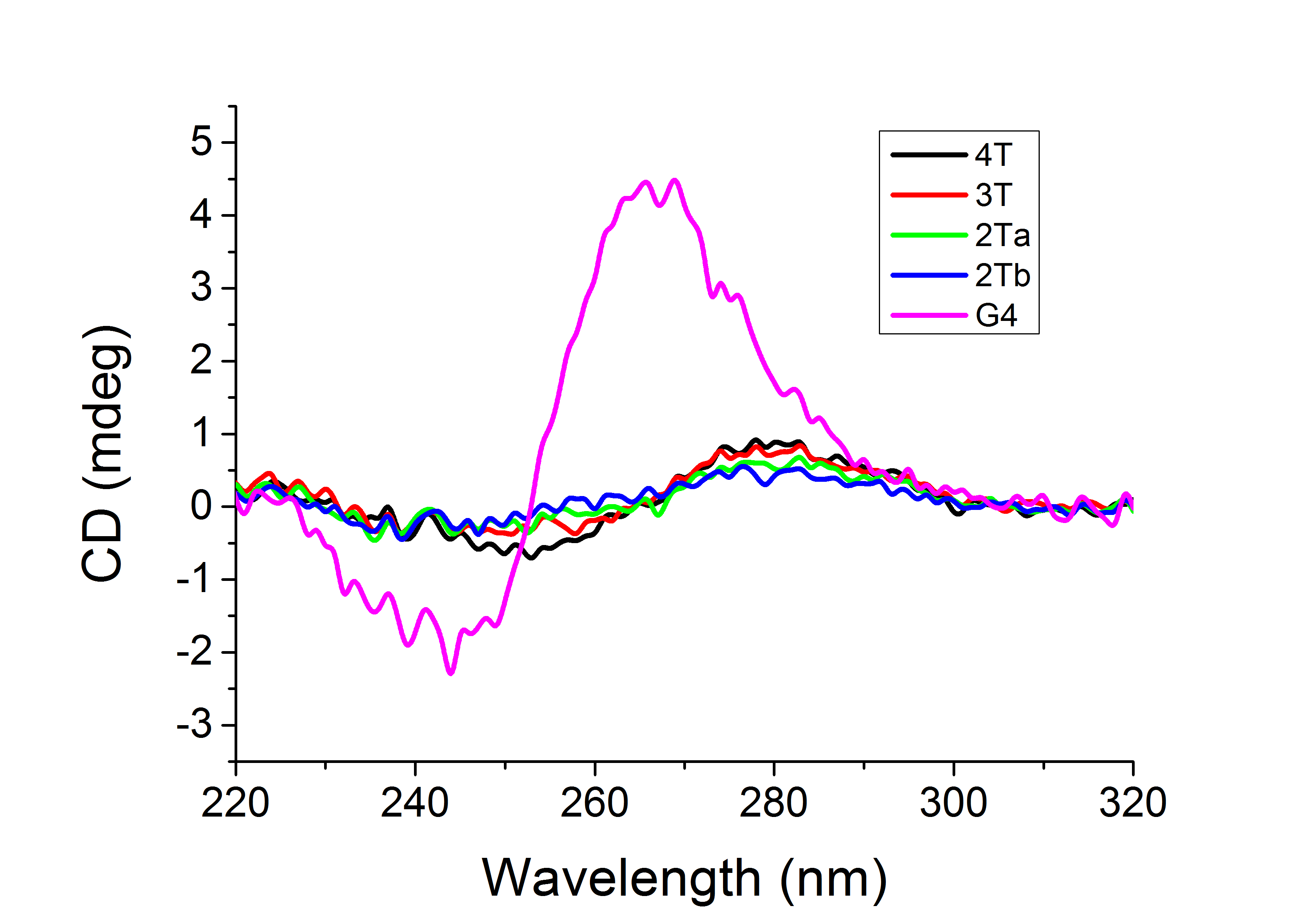


**Figure 6.** CD spectra of the used G-rich sequence (G4) and its analogous sequences (4T, 3T, 2Ta, 2Tb) in the presence of 10mM K^+^.


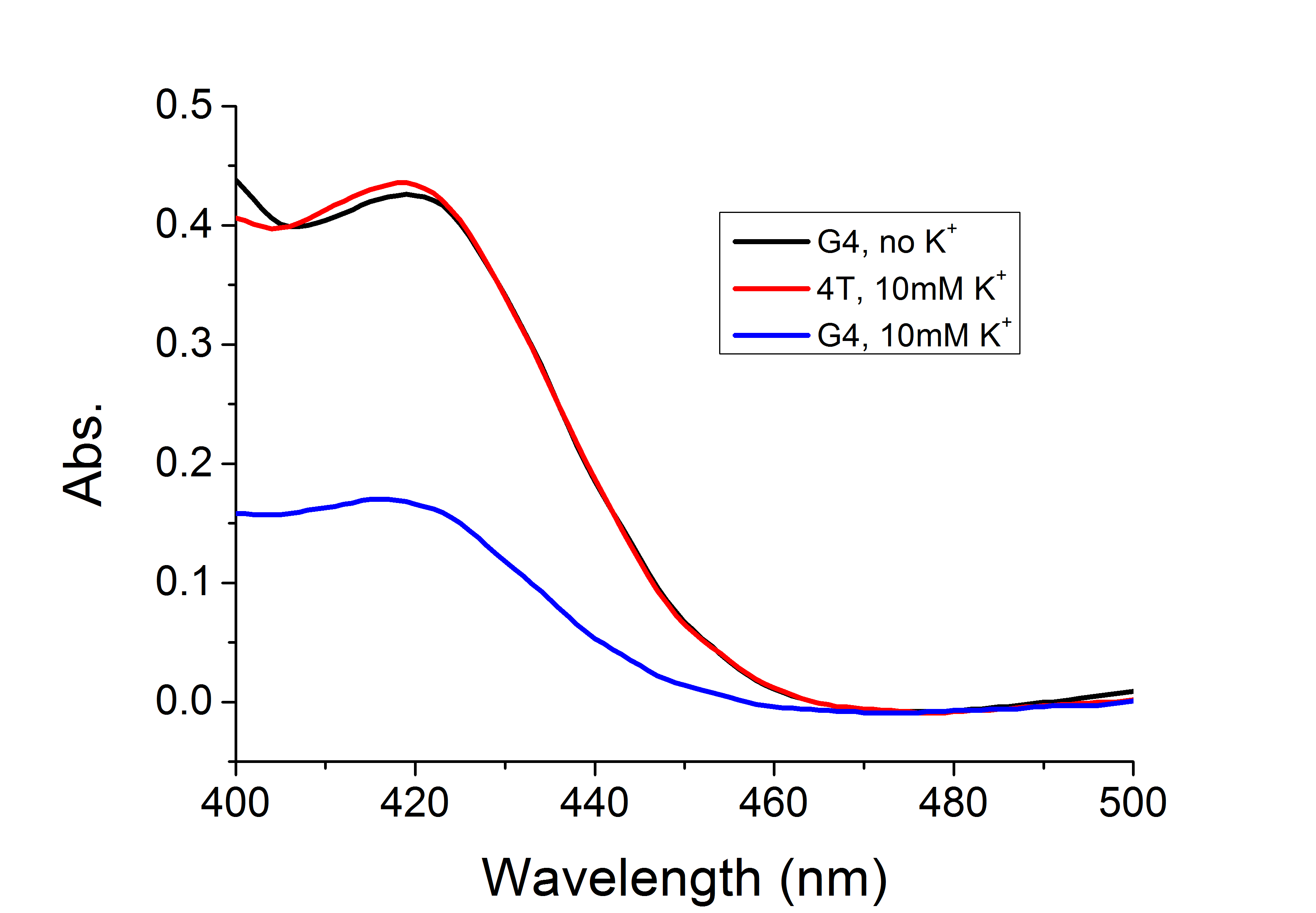

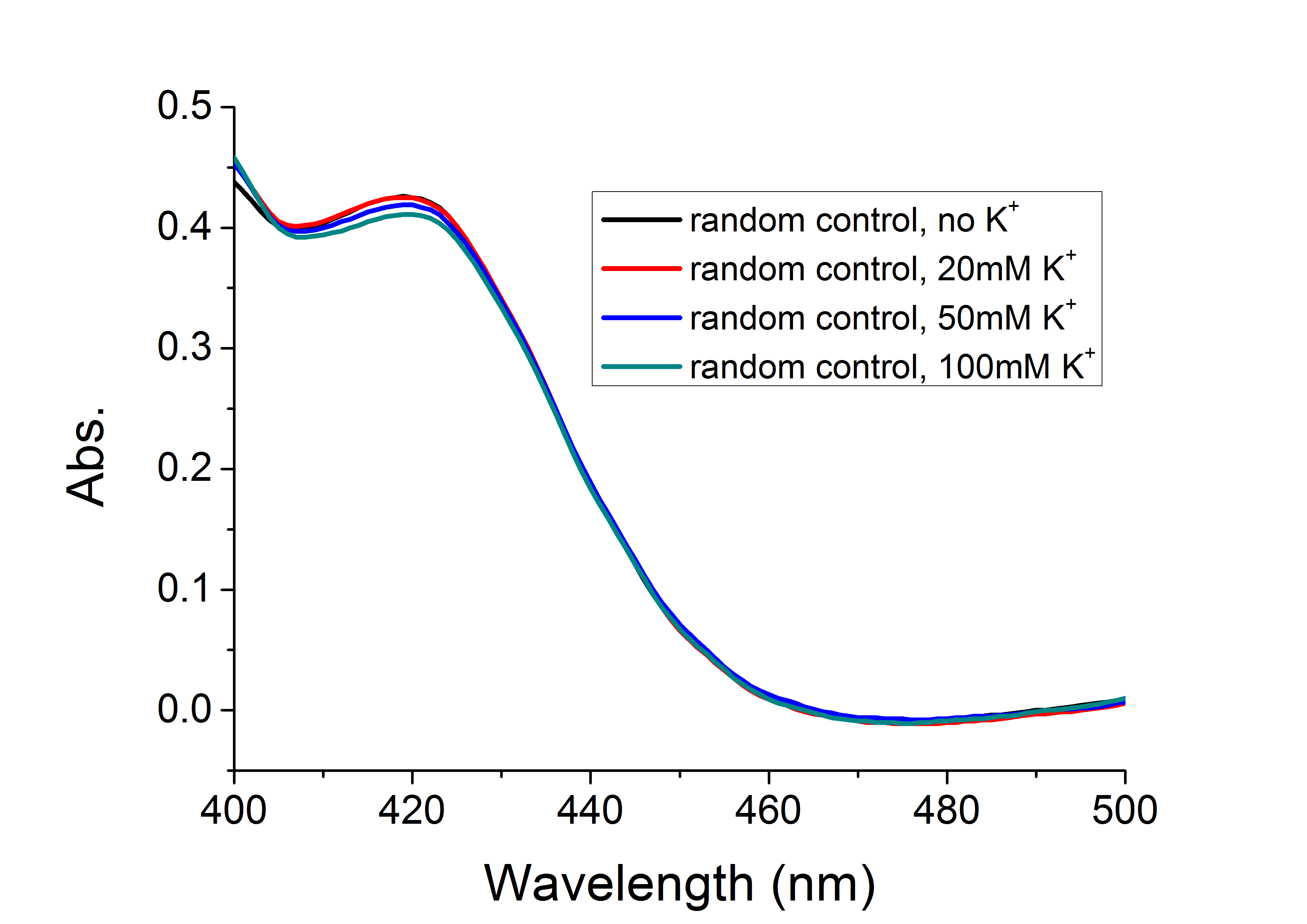


a

b

**Figure 7.** Catalytic activities of non K^+^ responsive DNA-immobilized AuNPs: (a) G4 and its analogous sequence 4T; (b) random control sequence.


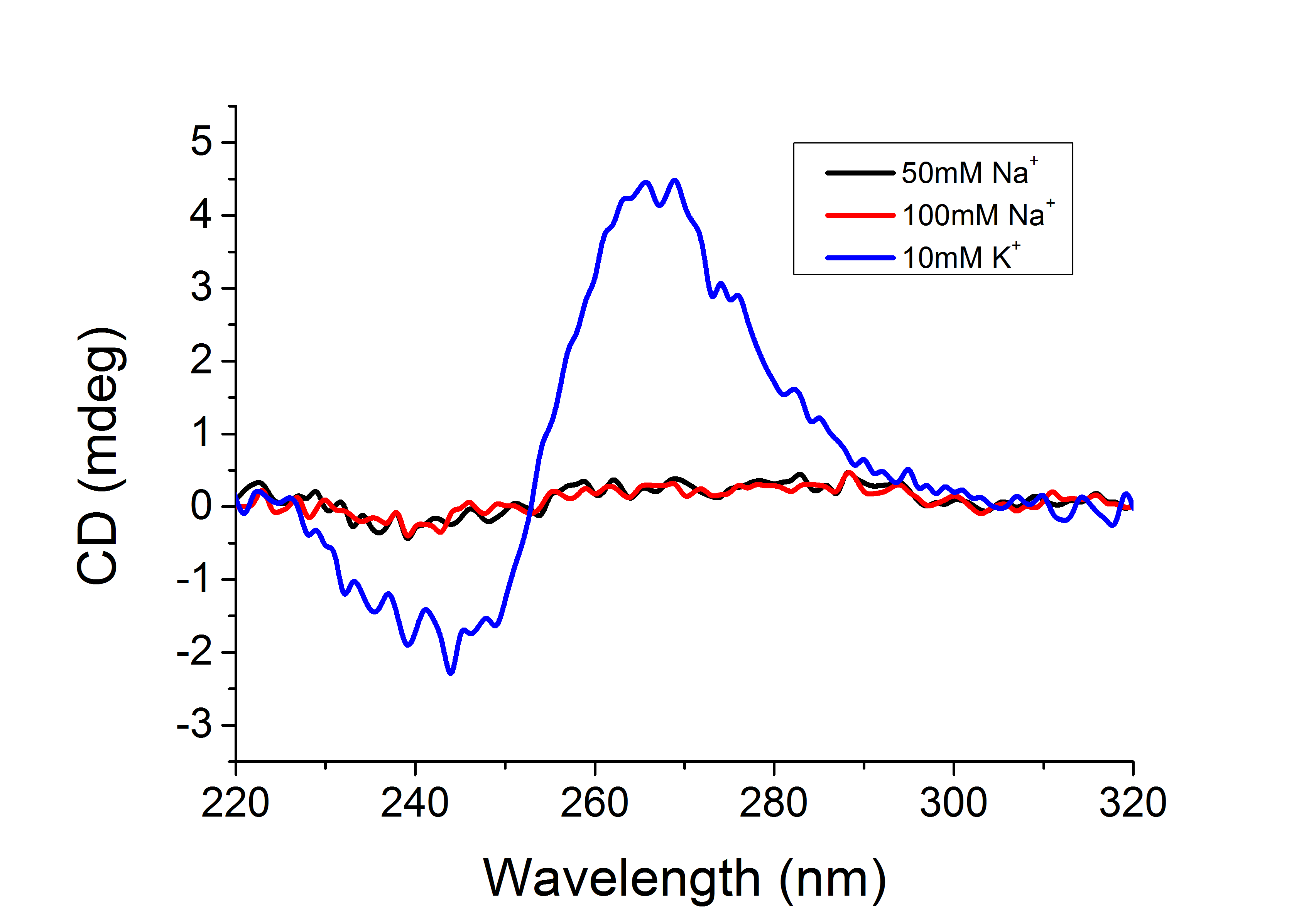

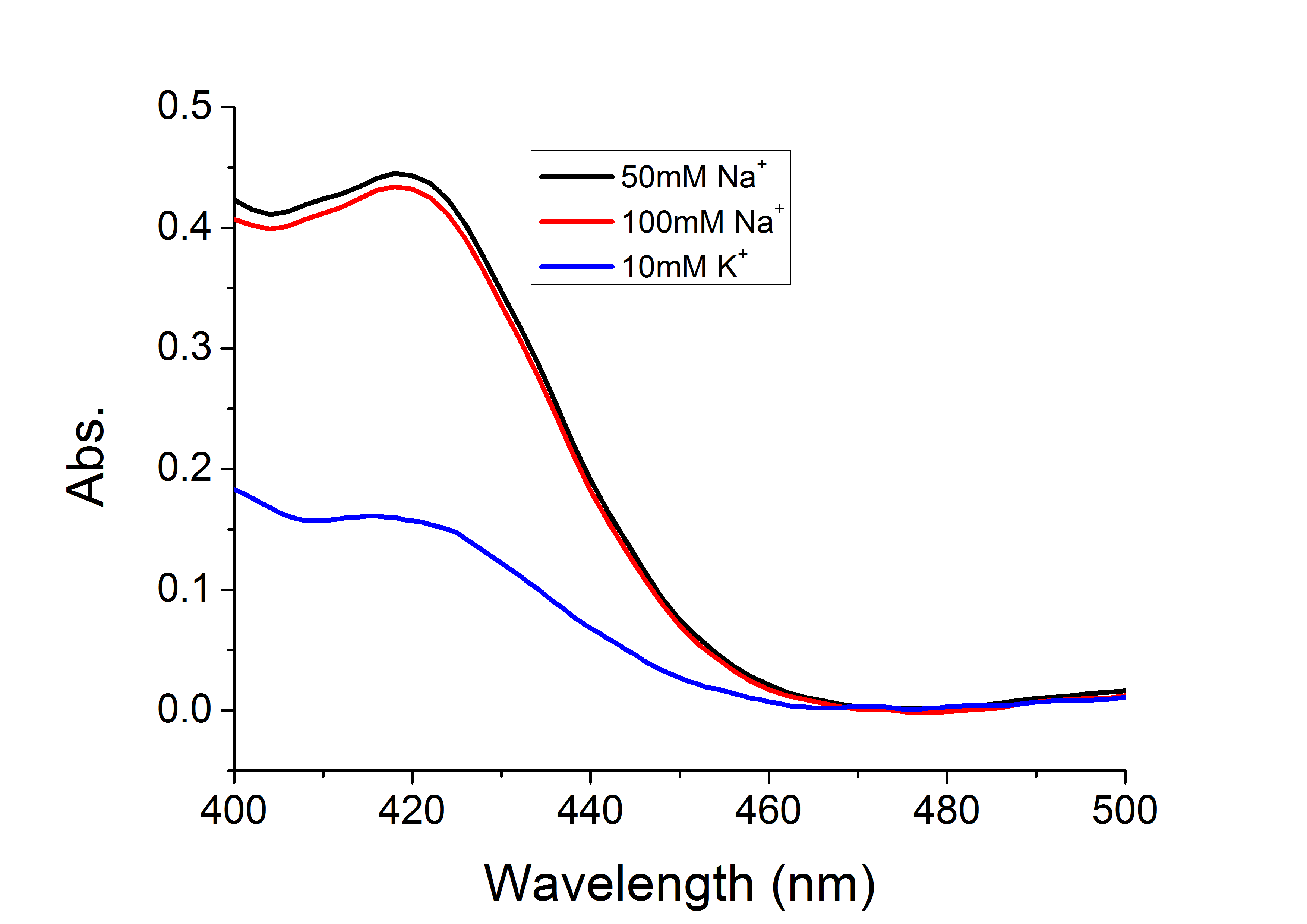


b

a

**Figure 8.** CD spectra (a) and catalytic activities (b) of the G-rich DNA (G4) in the presence of Na^+^.


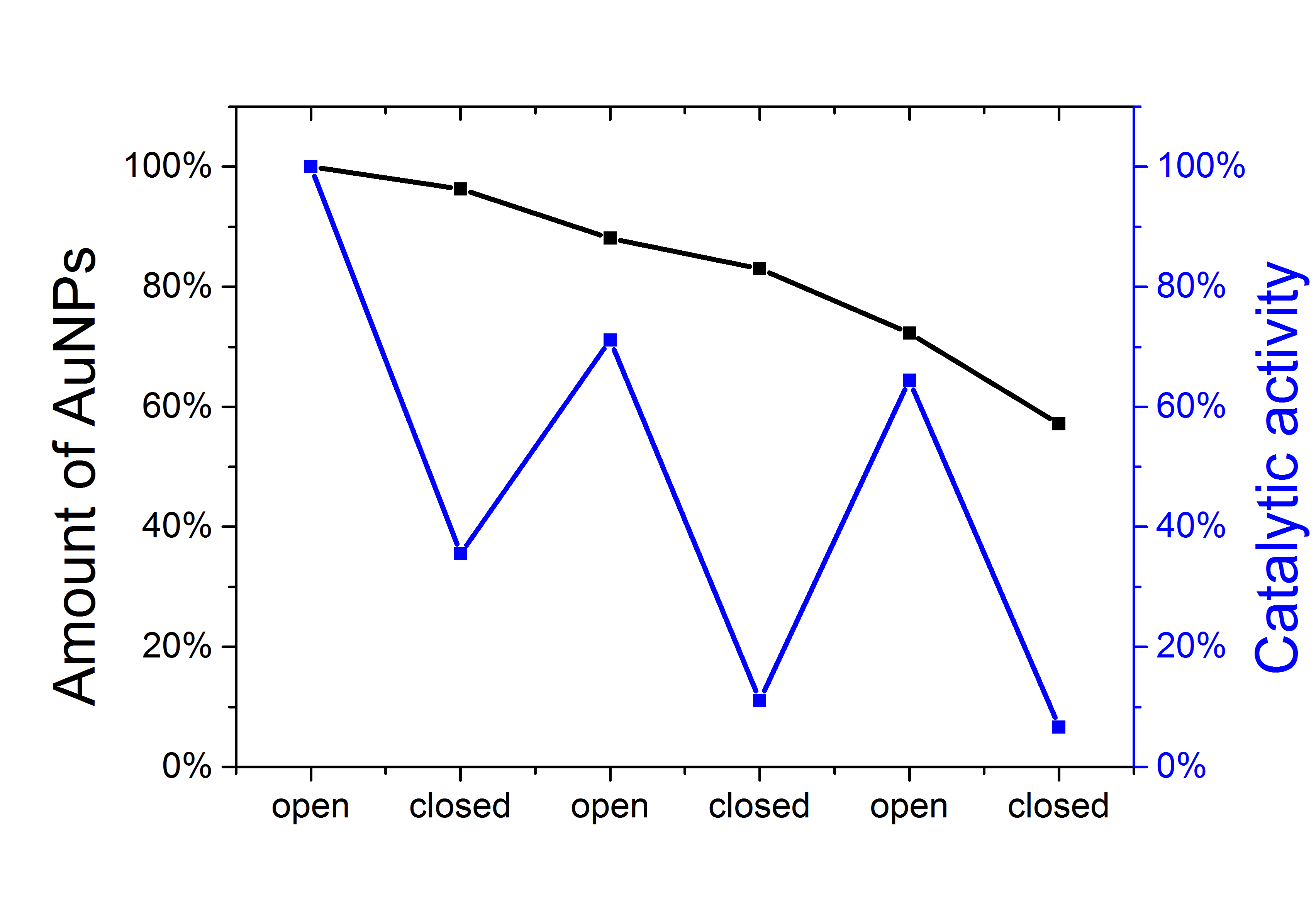


**Figure 9.** Comparison between the loss of AuNPs and the decay of the catalytic activity.
